# The Brain and the Nerves

**Published:** 1885-01

**Authors:** 


**The Brain and the Nerves: Their Ailments and their Exhaustion.** By THOMAS STRETCH DOWSE, M. D., Fellow of the Medical Society of London. New York: G. P. Putnam's Sons, 27 and 29 West Twenty-third street. 1884. Price, $1.50.

This work is a supplement to a paper on "Nervous Exhaustion," read four years ago by this author before the Medical Society of London, which was received at that time with great favor, both in England and America. The contents are divided into six chapters, in addition to the introduction, of which the first treats of the facts which have been established in connection with the physiology and pathology of the nervous system up to the present time. The second chapter treats of nervous energy and exhaustion. The third chapter treats of some of the ordinary symptoms of the neurasthemic. The fourth chapter is devoted to some forms of mental derangement and bodily ailment due to nervous exhaustion. Chapter five treats of the hereditary and nervous constitutions which are especially liable to exhaustion and fatigue. The sixth chapter is devoted to the treatment of nervous exhaustion. At this time, when medical men constantly meet with this class of diseases, the present work is a valuable one for study and reference.

